# Potential of Sorghum Seeds in Alleviating Hyperglycemia, Oxidative Stress, and Glycation Damage

**DOI:** 10.3390/molecules29153445

**Published:** 2024-07-23

**Authors:** Nora Ben El Mahdi, Laurent Lemée, Quentin Blancart Remaury, Lilian Eloy, Naima Nhiri, Naoufal Lakhssassi, Francesco Cacciola, Mohamed Nhiri

**Affiliations:** 1 Laboratory of Biochemistry and Molecular Genetics, Faculty of Sciences and Technologies of Tangier, BP 416, Tangier 90000, Morocco; 2 IC2MP-UMR CNRS 7285, Institut de Chimie des Milieux et Matériaux de Poitiers, University of Poitiers, 86073 Poitiers CEDEX, France; laurent.lemee@univ-poitiers.fr (L.L.); quentin.blancart.remaury@univ-poitiers.fr (Q.B.R.); lilian.eloy@univ-poitiers.fr (L.E.); 3 Institute for the Chemistry of Natural Substances, CNRS, Paris Saclay University, 91190 Gif-sur-Yvette, France; naima.nhiri@cnrs.fr; 4 School of Agricultural Sciences, College of Agricultural, Life and Physical Sciences, Southern Illinois University, Carbondale, IL 62901, USA; naoufal.lakhssassi@siu.edu; 5 Department of Biomedical, Dental, Morphological and Functional Imaging Sciences, University of Messina, 98125 Messina, Italy

**Keywords:** anti-glycation, reactive oxygen species, phytochemical composition, antioxidant enzymes, sorghum bicolor ecotypes seeds

## Abstract

Diabetes mellitus, characterized by dysregulated glucose metabolism, oxidative stress, and the formation of advanced glycation end products, poses a significant global health burden. In this study, we explored the potential of sorghum (*Sorghum bicolor*) seeds, known for their abundant phytochemical composition, as a natural remedy for diabetes and its associated damage. High-performance liquid chromatography/high-resolution mass spectrometry analysis revealed a remarkable phenolic richness in sorghum grains, including gallic acid, quercetin, and the predominant procyanidin B-1, with ecotype-specific variations in flavonoid distribution. Elemental analysis by ICP showed an abundance of macro-elements (Ca, K, Mg), trace elements (Fe, Mn, Si, Zn), and ultra-trace elements (B, Co, Cr, Cu, Mo, Se, V) essential for human health, supporting its therapeutic and nutritional potential. Additionally, the results demonstrated variable total phenolic contents (188–297 mg GAE/g dE) and total flavonoid contents (66–78 mg QE/g dE), with corresponding differences in antioxidant activities across the five ecotypes. Treatment with sorghum seed extract (SE1) significantly reduced oxidative stress markers, such as malondialdehyde (MDA)by 40% and hydrogen peroxide (H_2_O_2_) by 63%, in diabetic mice, compared to untreated diabetic controls. Moreover, sorghum extracts exhibited a remarkable increase in antioxidant enzyme activities, including a 50% increase in superoxide dismutase (SOD) activity and a 60% increase in glutathione peroxidase (GPx) activity, indicating their potential to bolster antioxidant defenses against diabetes-induced oxidative stress. These findings underscore the therapeutic potential of sorghum seeds in diabetes management and prevention, paving the way for the development of functional foods with enhanced health benefits.

## 1. Introduction

Diabetes mellitus, characterized by chronic hyperglycemia due to deficiencies in insulin secretion, action, or both, poses a significant global health concern, with 463 million adults affected in 2019 and projections indicating a rise to 700 million by 2045 [1]. Glycation, a non-enzymatic reaction between reducing sugars and proteins, lipids, or nucleic acids, leads to the formation of advanced glycation end products (AGEs), which contribute to diabetic complications such as nephropathy, retinopathy, neuropathy, and cardiovascular diseases [2].

Natural products, particularly plant-derived phenolic compounds, have shown promise in mitigating glycation and AGE formation while reducing the side effects of pharmaceutical drugs due to their antioxidant and anti-inflammatory properties [3,4]. Sorghum (*Sorghum bicolor*), a widely cultivated cereal crop, is rich in phenolic compounds and has demonstrated significant antioxidant, anti-inflammatory, and antidiabetic effects, making it a promising candidate for diabetes management [5]. Recent studies have highlighted the health-promoting properties of sorghum seeds [6,7].

This study aims to assess the therapeutic potential of ethanolic extracts from five different ecotypes of sorghum seeds in diabetes management, focusing on their ability to mitigate glycation and AGE formation. We will examine the phytochemical composition of the seed extracts, their antioxidant activity, and their anti-glycation properties both in vitro and in vivo using an alloxan-induced murine model of diabetes. By elucidating the bioactivity mechanisms of sorghum seed extracts, particularly their inhibition of glycation and AGE formation, this research aims to support the use of natural products in diabetes management and contribute to the development of novel dietary supplements or pharmaceutical agents for diabetes prevention and treatment.

## 2. Results and Discussion

### 2.1. Phytochemical Profile and Characterization of Bioactive Compounds Using HPLC-HRMS

Phenolic compounds are widely recognized for their health benefits, including antioxidant, anti-inflammatory, and anticancer effects. Sorghum (*Sorghum bicolor*) seeds, a staple cereal crop, are rich in phenolics and offer potential health benefits. Although several studies have examined the phenolic composition of sorghum seeds, comprehensive profiling using advanced techniques like high-performance liquid chromatography coupled with high-resolution mass spectrometry (HPLC-HRMS) is needed. This study aims to characterize the phenolic compounds in sorghum seed ethanolic extract using HPLC-HRMS and compare our findings with the existing literature.

**Figure 1:**
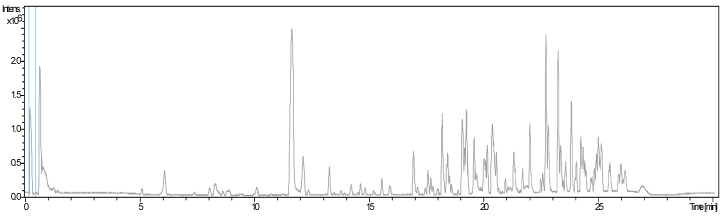
Chromatogram (HPLC-HRMS) of flavonoid compounds in seed ethanolic extract of sorghum ecotype 1.

HPLC-HRMS analysis (Table 1/Figure 1) revealed a rich array of phenolic compounds in sorghum seed ethanolic extract, consistent with previous studies. Identified phenolic acids include gallic acid, vanillic acid, protocatechuic acid, hydroxybenzoic acid, caffeic acid, and ferulic acid, demonstrating the diverse chemical composition of sorghum seeds. Flavonoids identified include procyanidin B-1, catechin, eriodictyol, taxifolin, naringenin, apigenin, quercetin, and rutin, enriching the phenolic profile of sorghum seeds. Our findings align closely with recent reports on sorghum phenolics using HPLC-HRMS and reinforce the robustness of our analytical approach [8,9,10,11]. Ecotype-specific variations observed are consistent with those reports, highlighting the importance of genetic and environmental factors in sorghum phenolic research [10,11]. 

**Figure 2:**
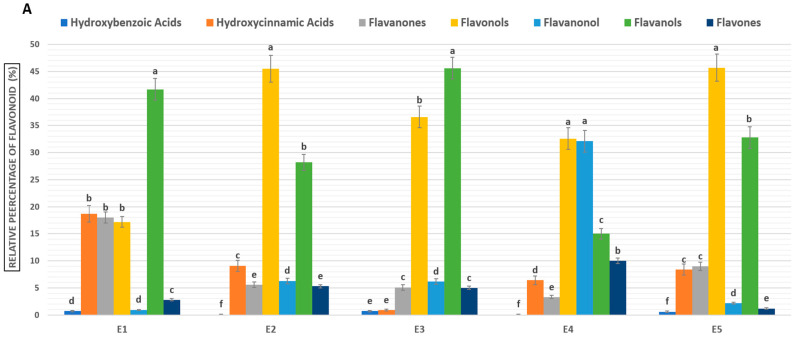

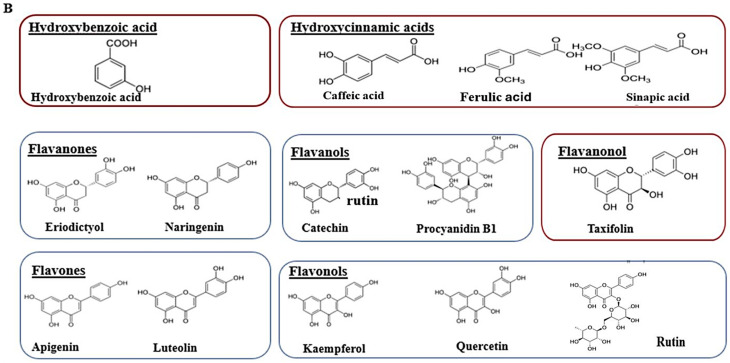
Compounds Identified by HPLC-HRMS in 5 sorghum seed ecotypes. (**A**) Relative percentage of identified compounds in the five sorghum ecotypes (E1, E2, E3, E4, E5), including hydroxybenzoic acids (I), hydroxycinnamic acids (II), flavanones (III), flavonols (IV), flavanonols (V), flavan-4-ols (VI), and flavones (VII). Letters above the bars indicate significant differences between compound classes for each ecotype separately according to ANOVA followed by Tukey’s HSD post hoc test, with significance letters assigned in descending order of means. (**B**) Chemical structures of the identified compound families.

The analysis of flavonoid composition across five sorghum ecotypes (Figure 2) reveals a diverse array of flavonoids. Procyanidin B-1, a condensed tannin with remarkable antioxidant properties, is the predominant compound. Variations in flavonoid distribution among ecotypes were observed: flavanols dominate in ecotypes 1 and 3, flavonols in ecotypes 2 and 5, and flavanonols in ecotype 4. These profiles underscore the interplay between genetic diversity and environmental factors in flavonoid biosynthesis. Other noteworthy flavonoids identified include catechin, quercetin, and kaempferol, all known for their health-promoting properties. Comparative analysis with the existing literature reaffirms the presence of these flavonoids in sorghum seeds, underscoring their potential antioxidant, anti-inflammatory, and anticancer benefits [11]. 

### 2.2. Elemental Analysis of Mineral Composition of Sorghum by ICP

Elemental analysis of sorghum seeds provides a detailed portrait of their mineral composition, offering valuable insights into the potential therapeutic benefits of this cereal crop. Across different ecotypes, significant variations in macroelements, microelements, and ultra-trace elements underscore the rich diversity of bioactive compounds in sorghum plants.

Macroelements, such as calcium (Ca), potassium (K), and magnesium (Mg), exhibit notable concentrations, with ecotype 1 particularly standing out with Ca levels at 199 mg/kg, K at 510 mg/kg, and Mg at 260 mg/kg (Table 2). These essential minerals are integral to fundamental physiological processes, including bone density, muscle contraction, and nerve signaling. The substantial abundance of macroelements in sorghum seeds positions this cereal as a natural reservoir of vital nutrients essential for overall health and vitality, consistent with recent findings [12,13].

Microelement variability further enriches sorghum’s therapeutic potential, with iron (Fe) concentrations reaching up to 57 mg/kg and manganese (Mn) up to 28 mg/kg. Silicon (Si) content spans from 361 to 472 mg/kg, while zinc (Zn) levels range from 12 to 33 mg/kg (Table 2). Iron and manganese are pivotal for oxygen transport and antioxidant defense mechanisms, respectively, while silicon and zinc contribute significantly to bone integrity and immune function. These microelements in sorghum seeds offer promising avenues for supporting cardiovascular health, bolstering immune resilience, and managing oxidative stress, as highlighted in previous studies [14,15].

The discovery of ultra-trace elements further underscores sorghum’s nutritional complexity and therapeutic promise. Boron (B) concentrations range from 1.09 to 2.15 mg/kg, cobalt (Co) is found in trace amounts (<100 µg/kg), chromium (Cr) levels span from 68 to 688 µg/kg, copper (Cu) ranges from 1.36 to 4.41 mg/kg, molybdenum (Mo) from 0.38 to 0.96 mg/kg, selenium (Se) from 12 to 23 µg /kg, and vanadium (V) from 16 to 397 µg/kg (Table 3). These ultra-trace elements are associated with various health benefits, including antioxidative properties, improved metabolic regulation, and modulation of thyroid function. Incorporating sorghum-based foods into the diet offers a holistic approach to harnessing these essential nutrients, thereby promoting overall well-being and vitality, as supported by recent investigations [16,17].

While the present study focused on the beneficial properties of sorghum seed extracts, such as their antioxidant and antidiabetic activities, it is crucial to consider the safety of these extracts for practical applications. ICP analyses revealed the presence of essential and trace elements, with the absence of potentially toxic elements at concerning levels, such as cadmium (Cd) and lead (Pb) (Table 2 and Table 3). However, while these results are reassuring, they are insufficient to assess the extracts’ overall toxicity. To ensure the safety of sorghum extracts, comprehensive toxicological studies should be conducted. These studies should include:

Evaluation of Acute and Chronic Toxicity: Acute and chronic toxicity studies on animal models to determine safe dosage ranges and identify potential short-term and long-term adverse effects.

Cytotoxicity Tests: In vitro cytotoxicity assays using various cell lines to assess potential toxic effects at the cellular level.

Monitoring of Side Effects: Monitoring potential side effects, including allergic reactions, gastrointestinal disorders, and liver or kidney toxicity.

Finally, although the data presented in Table 2 were obtained from single measurements without replicates, the observed values are consistent with the existing literature and remain reliable. Future studies should include multiple biological and technical replicates to ensure statistical robustness and validate these findings.

### 2.3. Total Phenolic and Flavonoid Content

The total phenolic and flavonoid content of sorghum’s ecotype extracts, as presented in Table 4, reveals insights into the phytochemical composition of sorghum seeds. The variations observed in polyphenols, flavonoids, and tannins across different ecotypes highlight the diversity of bioactive compounds present in sorghum plants.

When we compare our findings with the existing literature, we observe consistency in certain trends. For instance, ecotype 1 exhibited the highest extract yield, consistent with prior studies [18,19]. Regarding polyphenol content, ecotype 1 showed the highest concentration, while ecotype 2 had the lowest, aligning with previous reports [20,21].

Likewise, discernible differences in flavonoid content were observed among the various ecotypes, with ecotype 3 exhibiting the highest concentration and ecotype 2 demonstrating the lowest. This consistency with previous studies [22,23] suggests a pattern influenced by genetic and environmental factors.

Furthermore, tannin content varied among ecotypes, with ecotype 1 exhibiting the highest levels, followed by ecotype 4. Recent findings [22,23] support these trends, highlighting the multifaceted influence of genetic and environmental factors on sorghum’s phytochemical profile.

In conclusion, the analysis of total phenolic and flavonoid content underscores the need to consider genetic and environmental factors in determining the phytochemical composition of sorghum seeds. Further research should focus on elucidating the biosynthesis mechanisms of these bioactive compounds and their potential health benefits.

### 2.4. Antioxidant Activities

The antioxidant properties of sorghum ecotypes, as presented in Table 5, reveal their potential health benefits. Among the ecotypes, E4 exhibited the strongest DPPH scavenging activity, with the lowest IC_50_ value of 0.043 mg/mL, indicating robust antioxidant potential. Conversely, E2 showed the highest IC_50_ value of 0.06 mg/mL, suggesting relatively weaker antioxidant activity than the others. These findings align with previous research highlighting variability in sorghum antioxidant activity [11,22].

In addition to DPPH scavenging, ABTS assay results demonstrated E1’s potent activity of 0.090 mg/mL, contrasting with E5’s weaker activity of 0.140 mg/mL, consistent with prior studies [23].

Metal chelating activity, crucial for preventing oxidation reactions, varied among ecotypes. E5 exhibited the highest activity of 1.297 mg/mL, while E1 showed the lowest activity of 2.57 mg/mL, aligning with previous findings [22].

Reducing power, indicating electron donation ability, ranged from 90.16 mg AAE/g dE to 121.813 mg AAE/g dE. E5 displayed the highest and E2 the lowest, consistent with diverse antioxidant potentials.

Comparatively, Trolox, a well-known antioxidant standard, showed DPPH scavenging activity of 0.203 mg/mL, indicating that some sorghum ecotypes, like E4, possess competitive antioxidant potential. EDTA, used as a standard, showed activity of 0.189 mg/mL for metal chelating activity, suggesting that some sorghum ecotypes also have significant metal chelating capabilities.

The identified bioactive compounds (Figure 1, Table 1) can neutralize free radicals through electron transfer and proton donation reactions, reducing oxidative stress. Specific reactivity tests and metabolic pathway analyses can provide further insights into these mechanisms. Additionally, utilizing cellular models to observe the effects of sorghum extracts at the cellular level would help understand the changes in oxidative stress markers and the cellular signaling pathways involved in the antioxidant response

The diverse antioxidant capacities observed among sorghum ecotypes might significantly affect combating diabetes-related complications such as advanced glycation end products (AGEs) and reactive oxygen species (ROS) production. These antioxidant properties may help mitigate oxidative stress, a key factor in the progression of diabetes and its associated health issues. By scavenging free radicals and inhibiting ROS-mediated damage, sorghum antioxidants hold promise in alleviating diabetic complications, including cardiovascular diseases, nephropathy, and neuropathy. Sorghum antioxidants mitigate oxidative stress by enhancing the activity of key antioxidant enzymes such as superoxide dismutase (SOD), catalase, and glutathione peroxidase (GPx). These enzymes are crucial in neutralizing reactive oxygen species (ROS) and protecting cellular components from oxidative damage. Further research is needed to explore the specific mechanisms underlying how sorghum antioxidants interact with pathways related to AGE formation and ROS generation. Understanding these mechanisms will enable the development of targeted interventions using sorghum-based products to improve health outcomes in individuals with diabetes.

### 2.5. In Vitro Antidiabetic Activity: α-Amylase and α-Glucosidase Inhibition Effects

Sorghum-E extracts’ in vitro antidiabetic activity from five ecotypes was assessed using α-amylase and α-glucosidase inhibition assays. These enzymes are crucial for carbohydrate digestion and glucose absorption, making them prime targets for antidiabetic treatments. Sorghum extracts may reduce hyperglycemia by inhibiting the activity of carbohydrate-hydrolyzing enzymes such as α-amylase and α-glucosidase. By slowing down the breakdown of carbohydrates into glucose, sorghum extracts can help manage postprandial blood glucose levels, thereby acting as a natural approach to glycemic control. The inhibitory effects were quantified by determining IC_50_ values, representing the concentration of extract required to inhibit 50% of enzyme activity (Table 6).

The potent inhibitory activity of sorghum seed extracts against α-amylase and α-glucosidase enzymes suggests their promising role as a natural inhibitor for controlling postprandial blood glucose levels. This aligns with findings from recent studies investigating the antidiabetic properties of sorghum extracts [24,25,26,27]. These observations imply that sorghum seed extracts could potentially regulate carbohydrate digestion and absorption, contributing to glycemic control. Therefore, further exploration of sorghum-derived compounds as potential therapeutic agents for glycemic management and related metabolic conditions is warranted.

### 2.6. In Vitro Antiglycation Activities 

**Figure 3:**
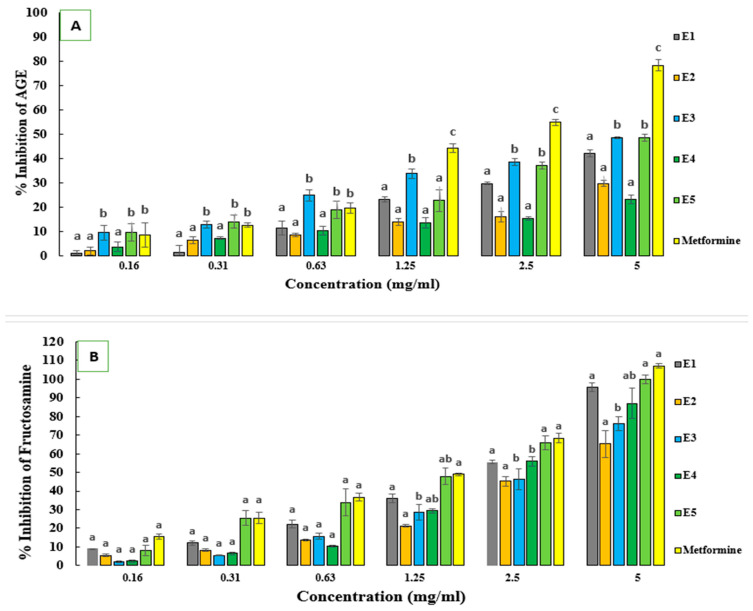
(**A**) Percentage of inhibition of AGE formation and (**B**) percentage of inhibition of fructosamine in the BSA/fructose system. Each value represents the mean of three replicates. Bars represent the standard error. Different letters indicate a significant difference between conditions of the same concentration at *p* < 0.05.

Figure 3A illustrates the percentage of inhibition of advanced glycation end products (AGEs) by different sorghum ecotypes across varying concentrations, alongside a positive control (Metformin). AGEs, formed through non-enzymatic glycation, contribute to numerous pathophysiological processes, notably diabetes and cardiovascular diseases. The bioactive compounds in sorghum, including flavonoids and phenolic acids, can inhibit the formation of advanced glycation end products (AGEs) by interacting with the glycation pathways. These compounds can bind to free sugars or amino groups in proteins, preventing the non-enzymatic reactions that lead to AGE formation. Additionally, they can act through several other mechanisms, such as neutralizing free radicals and reducing oxidative stress, which is a key driver in AGE formation. Sorghum bioactives can also neutralize reactive intermediates like methylglyoxal, a precursor in AGE formation, and chelate metal ions such as iron and copper, which catalyze the formation of AGEs.

Examining the inhibitory effects across different concentrations underscores dose-dependent responses. At lower concentrations (0.16 and 0.31 mg/mL), the sorghum ecotypes demonstrated modest inhibitory effects, with ecotypes 3 and 5 showing slightly higher inhibition. As concentrations increased, so did inhibitory activity against AGE formation. Ecotype 5 consistently exhibited the highest inhibition at higher concentrations (2.50 and 5.00 mg/mL). Compared with Metformin, sorghum extracts displayed comparable or superior inhibitory activity against AGE formation, suggesting their potential as anti-glycation agents. Variability in inhibitory activity among sorghum ecotypes may stem from differences in phytochemical composition. Sorghum contains various bioactive compounds, including phenolic acids and flavonoids, known for their antioxidant and anti-glycation properties. These differences in composition likely influence inhibitory activity against AGE formation. These findings highlight sorghum’s potential as a functional food with anti-glycation properties. Further research is needed to identify specific bioactive compounds responsible for inhibition and explore their mechanisms of action in mitigating AGE-related complications. Clinical studies are warranted to validate sorghum’s therapeutic potential in managing diabetes and other AGE-associated disorders.

Moreover, the IC_50_ values reported by Yilmaz et al. [28] for sorghum extracts (ranging from 13.93 to 63.00 µg/mL) are notably lower than the concentrations observed in our study. This indicates potentially higher potency of the sorghum extracts tested by Yilmaz et al. in inhibiting AGE formation than the sorghum ecotypes examined in our study.

Farrar et al. [29] also demonstrated that ethanolic extracts of certain sorghum bran varieties containing anthocyanins, luteolinidin, and apigeninidin significantly inhibited protein glycation. While our study did not specifically investigate these compounds, the presence of phenolic compounds in sorghum, including flavonoids, aligns with previous findings suggesting their role in inhibiting protein glycation.

In conclusion, sorghum emerges as a promising candidate in food chemistry, offering potent anti-glycation properties that merit further exploration. Understanding the mechanisms underlying sorghum’s effects and its specific bioactive compounds is essential for leveraging its potential in preventing chronic diseases.

Figure 3B presents the concentration of sorghum extract (ecotypes 1–5) and Metformin, along with their respective percentage inhibition on fructosamine levels. Fructosamine is a marker of glycemic control, reflecting average blood glucose. 

Across all ecotypes (1–5), as the concentration of sorghum extract increases, there is a notable increase in the percentage inhibition of fructosamine levels. This indicates a dose-dependent response, suggesting that higher concentrations of sorghum extracts lead to more significant inhibition of fructosamine, indicative of improved glycemic control. Among the different ecotypes, there are variations in the efficacy of fructosamine inhibition. For instance, ecotype five consistently exhibits the highest percentage of inhibition across all concentrations compared to other ecotypes.

This suggests that ecotype 5 may contain bioactive compounds with stronger anti-glycemic properties than other ecotypes. Ecotypes 1 and 3 also demonstrate significant inhibition of fructosamine, albeit to a lesser extent compared to ecotype 5. However, ecotypes 2 and 4 show comparatively lower inhibition percentages, indicating potentially lower concentrations of bioactive compounds associated with glycemic control.

The efficacy of sorghum extracts for glycemic control, particularly at higher concentrations, warrants comparison to Metformin, a widely used anti-diabetic medication [30,31]. Metformin remains the first-line therapy for type 2 diabetes, showing superior efficacy in lowering blood glucose levels and reducing complications. Nevertheless, concerns about Metformin’s long-term safety prompt the exploration of alternative therapies like sorghum extracts [32,33]. While sorghum extracts demonstrate promising anti-glycemic effects, especially at higher concentrations, Metformin consistently exhibits a high percentage inhibition of fructosamine across all concentrations, often comparable to or exceeding the inhibition observed with sorghum extracts. Further investigation is necessary to compare the bioavailability, safety profiles, and long-term effects of sorghum extracts and Metformin on glycemic control.

Our results, dose-dependent inhibition of fructosamine levels, are consistent with previous research demonstrating their anti-diabetic properties due to their rich bioactive compound content [34,35]. Different sorghum ecotypes may vary in efficacy, potentially due to variations in phytochemical profiles, necessitating further research for standardization [33,35].

### 2.7. In Vivo Antidiabetic Effect of Sorghum Seeds in Alloxan-Induced Diabetic Mice 

#### 2.7.1. Effect of Sorghum Seed Extract on Fasting Blood Glucose Level and Body Weight in Diabetic Mice

The experiment involved inducing severe hyperglycemia in mice through alloxan injection, resulting in initial blood glucose levels of 501 mg/dL for the diabetic non-treated group (Diabetic.NT) at week 0 (Table 7). Subsequent treatment with sorghum seed extract at doses of 125 mg/kg and 250 mg/kg led to a gradual decrease in blood glucose levels over three weeks, with levels dropping to 287.89 mg/dL and 165.54 mg/dL, respectively, by week 3. Notably, both doses of sorghum seed extract significantly reduced blood glucose levels compared to the diabetic non-treated group. Furthermore, the higher dose (+250 mg/kg) demonstrated a more substantial reduction in blood glucose levels compared to the lower dose (+125 mg/kg), indicating a dose-dependent response. Additionally, the standard antidiabetic drug glibenclamide exhibited a significant but comparatively lower reduction in blood glucose levels by week 3. These results suggest that sorghum seed extract, particularly at higher doses, effectively lowers blood glucose levels in alloxan-induced diabetic mice, potentially rivaling or surpassing the efficacy of glibenclamide. Further investigations comparing sorghum seed extract with other antidiabetic agents are warranted to fully elucidate its therapeutic promise and safety profile.

#### 2.7.2. Effect of Sorghum Seed Extract on Biochemical Parameters in Diabetic Mice

**Figure 4:**
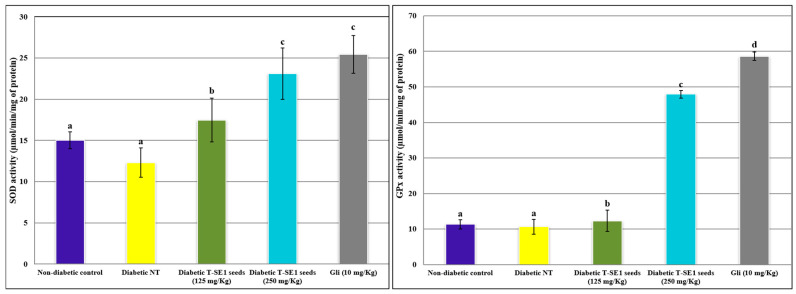

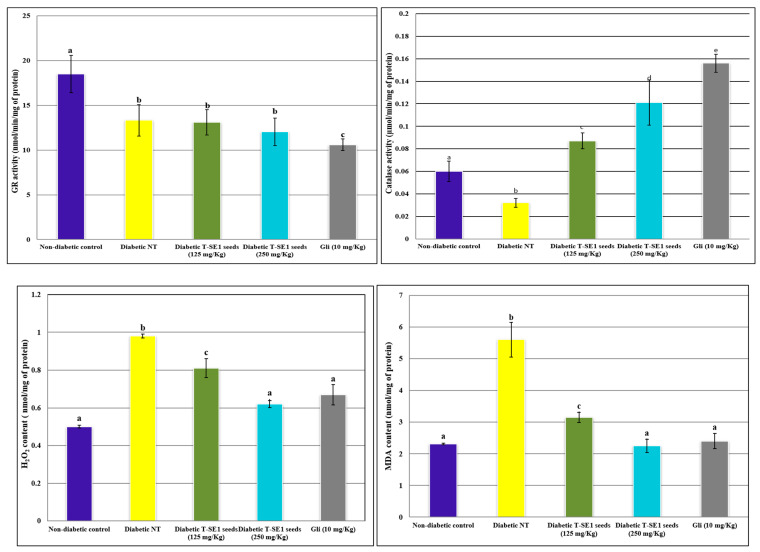
Effect of SE1 seeds on the activities of antioxidant enzymes in the liver of alloxan-induced diabetic mice. Abbreviations are SOD: superoxide dismutase; CAT: catalase; GR: glutathione reductase; GPx: glutathione peroxidase; MDA: malondialdehyde; H_2_O_2_: hydrogen peroxide. Bars represent the standard error. Different letters indicate significant differences among treatments at *p* < 0.05.

Figure 4 presents the impact of sorghum seed extract (ecotype 1) (SE1) on key biochemical parameters in alloxan-induced diabetic mice, juxtaposed against non-diabetic control mice and untreated diabetic counterparts (Diabetic NT). 

The levels of malondialdehyde (MDA) and hydrogen peroxide (H_2_O_2_) content in different treatment groups are as follows: In the NT diabetic group, elevated levels of MDA and H_2_O_2_ content are observed compared to the non-diabetic control, indicating increased oxidative stress associated with diabetes.

Treatment with sorghum seed extract at both doses shows a significant reduction in MDA and H_2_O_2_ content compared to the NT diabetic group. This suggests a potential antioxidative effect of sorghum seed extract in mitigating oxidative stress induced by diabetes. Interestingly, the reduction in MDA and H_2_O_2_ content appears to be dose-dependent, with the group treated with a higher dose (250 mg/kg) exhibiting a more pronounced decrease in oxidative stress markers compared to the group treated with a lower dose (125 mg/kg). Moreover, the antioxidative effect of sorghum seed extract seems comparable to that of glibenclamide, a standard antidiabetic medication, particularly at the higher dose. This suggests that sorghum seed extract may possess antioxidative properties that could contribute to its potential therapeutic benefits in diabetes management. Sorghum extracts may exert anti-inflammatory effects by modulating signaling pathways such as NF-κB and MAPK, which are involved in the inflammatory response. By reducing inflammation, sorghum extracts can further alleviate oxidative stress and protect against complications associated with chronic inflammation in diabetes. 

Comparisons with the existing literature on the antioxidative effects of sorghum seed extract in diabetic animal models could provide further insights into the findings and validate its potential as a natural antioxidant therapy for diabetes-related oxidative stress. For instance, studies by Wu et al. (2011) and Ajiboye et al. (2013) reported similar reductions in MDA and H_2_O_2_ levels following treatment with sorghum seed extract in diabetic rats, supporting our observations [36,37]. However, further investigations are warranted to elucidate the underlying mechanisms and establish the efficacy of sorghum seed extract as an adjunct therapy for diabetes [38]. The presence of specific bioactive compounds like procyanidin B-1 in sorghum contributes significantly to its antioxidant and antiglycation activities. Procyanidin B-1 is known for its ability to scavenge free radicals and inhibit protein glycation, highlighting its potential therapeutic benefits in managing diabetes and its complications.

Superoxide Dismutase (SOD) Activity: Diabetic mice exhibited diminished SOD activity compared to non-diabetic controls, corroborating previous studies indicating oxidative stress in diabetes. Notably, SE1 seed treatment, particularly at 250 mg/kg, resulted in a significant increase in SOD activity, aligning with previous findings showcasing the antioxidant potential of sorghum extracts in alleviating oxidative stress in diabetic models.

Catalase Activity: Reduced catalase activity observed in diabetic mice underscores impaired antioxidant defenses linked to diabetes-induced oxidative stress, consistent with prior research. SE1 seed treatment exhibited a dose-dependent elevation in catalase activity, akin to recent findings of Xiong et al. [39], suggesting the antioxidative prowess of sorghum extracts in bolstering antioxidant defenses against diabetic-induced oxidative stress.

Glutathione Peroxidase (GPx) Activity: A decline in GPx activity in diabetic mice reflects compromised antioxidant capacity, corroborating studies implicating oxidative stress in diabetes progression. SE1 seed treatment, especially at 250 mg/Kg, significantly elevated GPx activity, mirroring the findings of Li et al. (2020) [40], highlighting the potential dose-dependent influence of sorghum extracts in enhancing antioxidant defenses against diabetic-induced oxidative stress.

Glutathione Reductase (GR) Activity: Reduced GR activity in diabetic mice suggests perturbed redox homeostasis associated with diabetes, consistent with the literature on diabetic complications. While SE1 seed treatment showed potential in mitigating oxidative stress, further research is needed to fully elucidate its effects on GR activity and its contribution to antioxidant defenses in diabetic conditions, aligning with the call for comprehensive investigation in diabetic models [41] by Johnson et al. (2022).

In summary, the outcomes underscore the potential of sorghum seed extract (SE1) in mitigating diabetic-induced oxidative stress through modulation of crucial antioxidant enzyme activities, with findings resonating with the existing literature. However, further research is warranted to elucidate the specific bioactive compounds responsible for these effects and to explore SE1’s therapeutic potential in managing diabetic complications.

The results presented highlight the potential of sorghum seed extract as a natural therapeutic agent for managing diabetes and related complications, offering insights into its mechanisms of action and comparative efficacy against conventional treatments. The inhibitory activity of sorghum extracts against α-amylase and α-glucosidase enzymes, essential for carbohydrate digestion and glucose absorption, demonstrates their role in regulating postprandial blood glucose levels. These findings align with the recent literature, affirming sorghum’s antidiabetic properties attributed to its rich bioactive compound content.

Furthermore, this study elucidates sorghum’s anti-glycation properties, evidenced by its inhibitory effects on AGE formation. The dose-dependent response underscores sorghum’s potential as an anti-glycation agent, with certain ecotypes exhibiting superior inhibitory activity. Notably, sorghum extracts display comparable or superior efficacy to conventional medications like Metformin, warranting further investigation into their therapeutic potential and safety profiles.

Additionally, the antioxidative effects of sorghum seed extract offer promising avenues for combating oxidative stress associated with diabetes. The significant reductions in oxidative stress markers and enhancement of crucial antioxidant enzyme activities underscore sorghum’s role in bolstering antioxidant defenses. These findings are consistent with previous research, validating sorghum as a natural antioxidant therapy for diabetes-related oxidative stress.

The results of this study indicate that sorghum seed extracts possess significant antioxidant and anti-glycation properties, suggesting their potential as ingredients in functional foods. Incorporating these extracts into food products could provide benefits for managing diabetes and preventing associated complications. For example, sorghum-based products enriched with these extracts could be developed to help regulate blood glucose levels and reduce oxidative stress in diabetic patients. Moreover, the nutritional and therapeutic properties of sorghum seeds could be exploited to create dietary supplements aimed at improving overall metabolic health. These developments would not only diversify food options for individuals with diabetes but also promote healthier eating habits in the general population.

## 3. Materials and Methods

### 3.1. Plant Material and Extraction

**Figure 5:**
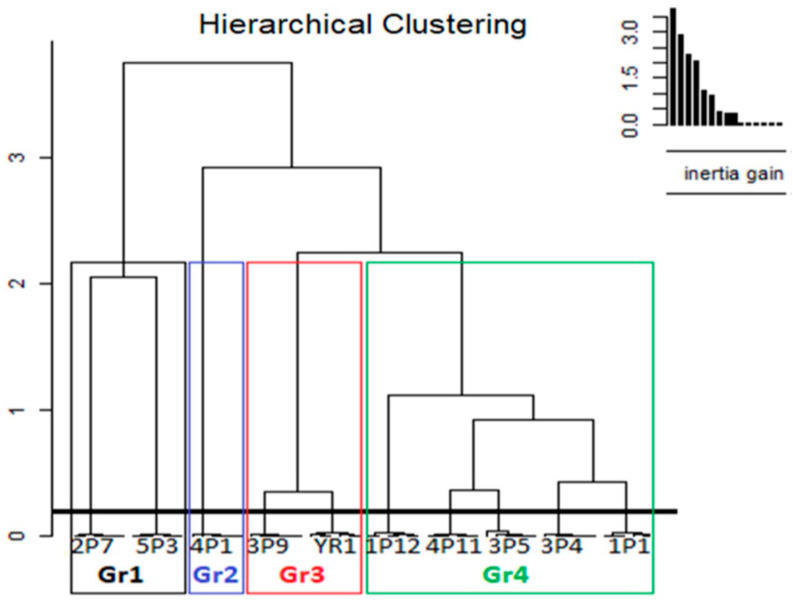
Hierarchical clustering of the 5 Moroccan sorghum seed ecotypes based on agromorphological traits.

The plant material consisted of 5 sorghum ecotypes (Figure 5) selected from a collection of 10 sorghum ecotypes [42] by Bouargalne et al. (2022), conserved at Laboratory of Biochemistry and Molecular Genetics, Faculty of Sciences and Technologies, Abdelmalek Essaadi University Tangier (Morocco).

This core collection is representative of the Moroccan cultivated sorghum varieties, which were sampled based on geographical origin. The study was conducted in Tinghir (17°25′ N latitude and 78° E longitude and minimum and maximum temperatures of 10 °C and 45 °C with an annual total rainfall of 619 mm) during 2015 summer season. Sowing was performed following a randomized complete block design (RCBD) with three replicates in 8 m rows with a row spacing 60 cm apart and plant-to-plant spaced at 20 cm. Standard cultivation practices followed locally were adopted. NPK fertilizer was applied at the rate of 100 kg/ha. Data on the morpho-agronomic traits were collected from six randomly selected individual plants for each ecotype. Meanwhile, the collected mature seeds were used for further biochemical experiments. The 25 morpho-agronomic traits that were measured or scored are described in Appendix A (Table A1) [42]. The whole seeds were washed with distilled water, shade dried, and powdered with a mechanical grinder; the powder was stored in an airtight container until needed for further use. Five distinct ecotypes (E1, E2, E3, E4, and E5) (Table 8) were subjected to extraction using 80% ethanol. The resulting homogenate underwent a 24 h incubation in darkness under continuous agitation at 260 rpm. Subsequently, the extract was filtered through Whatman filter paper and centrifuged at 5000× *g* for 10 min. Finally, evaporation at 40 °C yielded extracts with the following respective percentages for E1, E2, E3, E4, and E5: 26.73%, 17.05%, 22.49%, 25.23%, and 19.12%.

To ensure the long-term stability and reusability of the extracts, stability tests were conducted by storing the extracts at 4 °C and at room temperature. The chemical composition and antioxidant activity of the extracts were monitored over a period of six months. Additionally, reusability tests were performed by subjecting the extracts to multiple cycles of use in various applications, such as food preservation and pharmaceutical formulations, to evaluate their practical viability

### 3.2. HPLC-HRMS

HPLC-HRMS analysis was conducted utilizing a Thermo Scientific Ultimate 3000 (Thermo Fisher Scientific, Waltham, MA, USA) system coupled with a Bruker Impact HD Series Quadrupole-Time of Flight (QTOF) (Bruker, Billerica, MA, USA) mass spectrometer equipped with an Electrospray (ESI) ion source. The chromatographic separation was performed on an Acquity UPLC^®^ ® (Waters Corporation, Milford, MA, USA) BEH 1.7 µm column (100 × 2.1 mm), with a mobile phase flow rate of 0.4 mL min-1 and the column temperature maintained at 30 °C. The mobile phase consisted of ultrapure water (eluent A) and methanol (eluent B) containing 0.1% formic acid. Following the injection of a 10 μL sample, the gradient began with 1% B and remained constant for 2 min. Subsequently, it ramped up to 99% B over 23 min and remained constant for 2 min.

For the mass spectrometry method, the ESI source operated in positive mode with the following parameters: gas temperature of 200 °C, drying gas flow rate of 8.0 L min^−1^, nebulizer pressure of 2.1 Bar, capillary voltage of 2500 V, and a mass range from 50 to 1000 m/z.

### 3.3. Elemental Analysis by ICP

#### 3.3.1. Preparation Sample

Approximately 300 mg of the sorghum kernel ground to a fine and homogenized powder was weighed into a Teflon vessel. Two digestions were realized for each sample with different acid mixtures, 8.0 mL HNO_3_ and 7.0 mL HNO_3_ + 1.0 mL HF for silicon analysis were added then the vessel was sealed. An Anton Paar Multiwave (Graz, Austria) pro fitted with a rotor for 8 pressure vessels NXF100 was used. The temperature and pressure limit in the digestion program was fixed at 230 °C and 60 bars. The digestate was made up to a volume of 50 mL using de-ionized water. De-ionized 18 MΩ.cm^−1^ water used for the preparation of all blanks, standards, and sample solutions was obtained from a Millipore water purification system (Merck Millipore, Burlington, MA, USA). External calibration curves were performed using certified calibration standard solutions and diluted according to the working ranges required.

#### 3.3.2. Major and Trace Elements Using ICP-OES

Quantification of major (K, Mg, P, S) and minor (Ca, Fe, Mn, Si, Zn) elements was performed by inductively coupled plasma optical spectrometry (ICP-OES; Agilent 5110 VDV, Agilent Technologies, Santa Clara, CA, USA). The sample introduction system was constituted of a Seaspray™ nebulizer (Glass Expansion, Port Melbourne, Victoria, Australia) and a quartz double-pass cyclonic chamber. The analyses were conducted at IC2MP-UMR CNRS 7285, Institute of Chemistry of Environments and Materials of Poitiers, University of Poitiers, 86073 Poitiers CEDEX, France For silicon, an inert spray chamber was used to facilitate the analysis of HF digests without prior neutralization and avoid quartz sample introduction attacks from free HF. The parameter settings were as follows: Argon (Ar) plasma gas flow, 12 L.min^−1^; Ar auxiliary gas flow, 0.7 L.min^−1^; Ar nebulizer gas flow, 0.70 L.min^−1^; and radio frequency power 1200 W.

To avoid most spectral interferences, two wavelengths for each element were checked. An internal standard (ISTD) solution containing 1 mg/L of yttrium was delivered online to the sample before nebulization using a Y-connector. ISTD allows us to check and correct non-spectral interference.

#### 3.3.3. Ultra-Trace Elements Using ICP-MS

Quantification of trace elements (11B, 111Cd, 59Co, 52Cr, 63Cu, 95Mo, 23Na, 60Ni, 208Pb, 78Se and 51V) was performed by inductively coupled plasma mass spectrometry (ICP-MS Agilent 7800). Operating conditions are shown in Table 9. Spectrometer parameters were optimized daily using a 1 µg/L Agilent tuning solution. The spectrometer was equipped with a collision reaction cell (CRC) to reduce interferences. Selenium 78 was analyzed with hydrogen gas in CRC, and H_2_ flow gas rate was optimized to eliminate 38Ar40Ar interference. An additional interference on Se from rare-earth element doubly charged (156Gd^2+^) was avoided by adding 156Gd in the analyte list and calculating the applied Gd^2+^/Gd ratio correction. Lead was measured with the sum of the 3 most abundant isotopes 206, 207, and 208.

### 3.4. Total Phenolic Content

The total phenolic content was determined by the Folin–Ciocalteu (FC) method, following Singleton and Rossi [43] with some modifications [44]. Briefly, 100 µL of the sample solution was mixed with 400 µL of FC reagent and 1 mL of 7% sodium carbonate (Na_2_CO_3_) solution. The final volume was adjusted to 1.6 mL with distilled water. The reaction mixture was incubated in the dark for 30 min, followed by absorbance measurement at 725 nm using a spectrophotometer (EPOCH, BioTek) against a blank. The TPC of the extracts was expressed as milligrams of gallic acid equivalents per gram of dry weight (mg GAE/g dw) using a calibration curve generated with gallic acid as the standard. 

### 3.5. Total Flavonoid Content

The flavonoid content was assessed following the method of Huang et al. [45] with some modifications. Briefly, 40 µL of each sample was mixed with 10 µL of acetate potassium (1 M) and 10 µL of aluminum chloride (10%). Next, 100 µL of methanol 50% was added and the total volume was made up to 400 µL with distilled water. The absorbance of the mixture was taken at 415 nm. Quercetin was used as standard. The flavonoid content was expressed as milligrams of quercetin equivalence (QE) per gram of extract.

### 3.6. Tannin Content

The TTC was determined using the Folin–Denis method, with modifications. Briefly, 100 µL of extracts (1 mg/mL) was added to 500 µL of (1:10) Folin–Denis reagent. After 5 min, 400 µL of Na_2_CO_3_ 7.5% (*m*/*v*) was added. After 30 min of incubation, absorbance at 760 nm was measured (VARIAN Cary 50 UV-Vis). The standard was prepared using different concentrations of tannic acid. Results were reported in Tannic Acids Equivalents (TAEs) per g of sample.

### 3.7. Antioxidant Activities

#### 3.7.1. DPPH Radical Scavenging Assay

The radical scavenging ability of the extracts was monitored using the stable free radical DPPH (2,2-diphenyl-1-picrylhydrazyl) following the method described by Hatano et al. [46] with some modifications. Different concentrations of samples prepared in methanol solution with a serial dilution starting from the concentration of 1 mg/mL to 0.625 mg/mL were mixed with freshly prepared DPPH solution. The mixture was shaken vigorously and left to stand in the dark and at room temperature for 30 min. The reduction of the DPPH radical was measured at 517 nm. The DPPH scavenging activity was determined by calculating the percentage of DPPH discoloration using the following equation:

(*ADPPH* − *AS*) % *Scavenging effect* = [*ADPPH*] × 100

where AS is the absorbance value of the sample and ADPPH is the absorbance of the DPPH solution. The extract concentration providing 50% inhibition (IC_50_) was calculated from the graph of the scavenging effect percentage against the extract concentration in the solution.

#### 3.7.2. Radical Scavenging Activity against the Radical ABTS+

The radical scavenging activity against the radical ABTS+ was determined according to the method of Re et al. [47] with minor modifications as reported earlier [48].

#### 3.7.3. Metal Chelating Activity

As described by Dinis et al. [49], the ferrous ion chelating potential was evaluated using a reaction mixture of a panel of concentrations from each extract and FeCl_2_ (0.6 mM). The reaction was initiated by the addition of 5 mM ferrozine, and the absorbance was read after 10 min at 562 nm. The sample was replaced by methanol in the control. The IC_50_ was calculated as described earlier [44]. 

#### 3.7.4. Reducing Power Assay (FRAP)

The reducing power was evaluated following the Oyaizu method with some modifications [50]. Briefly, 100 µL of the sample extract was added to 250 µL of phosphate buffer (0.2 M, pH 6.6) and 500 µL of potassium ferricyanide (1%). After 20 min at 50 °C, 240 µL of trichloroacetic acid (10%) was added to each tube, and the mixtures were centrifuged at 3000 rpm for 10 min. Next, 100 µL of the supernatants was added to 100 µL of (dH_2_O) and 0.1 mL of FeCl_3_ (0.1%). The absorbance of all solutions was measured at 700 nm. Values are presented as mg of ascorbic acid equivalent per g dry weight (mg AAE/g dw).

### 3.8. Antiglycation Activities

#### 3.8.1. AGE Formation

The glycated BSA formation was performed according to a previously published method with some modifications [51]. In brief, BSA (10 mg/mL) was incubated with 0.5 M fructose in 0.1 M phosphate buffer (PB), pH 7.4, containing 0.02% sodium azide in the dark at 37 °C for 28 days. Before incubation, sorghum seed extract and Metformin were dissolved in PB and added to the mixtures. The fluorescent AGE formation of glycated BSA was determined using a spectrofluorometer at excitation and emission wavelengths of 355 nm and 460 nm, respectively. Metformin was used as a positive control in this study. The percentage of inhibition was calculated by the following equation:

inhibition (%) = [1 − ((FLs − FLsb))/((FLc − FLcb))] × 100

where FLs refers to the fluorescence intensity of the mixture, FLsb refers to the fluorescence intensity of sample blank (without fructose), FLc refers to the fluorescence intensity of the control mixture, and FLcb refers to the fluorescence intensity of the control blank mixture.

#### 3.8.2. Determination of Fructosamine

The fructosamine assay was tested after four weeks of incubation. Fructosamine reduces NBT and produces coloration, absorbing at 530 nm [52]. Briefly, 20 µL of the glycated materials was taken up, and 100 µL of 0.3 mM of NBT was added. The absorbance of the mixture was determined at 530 nm after an incubation step at 37 °C for 30 min.

Inhibition of fructosamine by sorghum seeds (%) = (Ai − Aj)/Ai × 100

where Ai represents the absorbent value of the control mixture and Aj corresponds to the glycation system in the presence of the inhibitor.

### 3.9. In Vitro Antidiabetic Enzymatic Assays 

#### 3.9.1. α-Amylase Inhibition Assay

The α-amylase inhibitory activity was measured according to methods described by [53,54] with some modifications. A 100 µL sample solution was premixed with 100 µL of α-amylase solution (0.1 U/mL in the pH 6.9 buffer) and incubated at 37 °C for 30 min. After pre-incubation, 100 µL of starch solution (0.25%) in the pH 6.9 buffer was added to each tube to start the reaction. The reaction was carried out at 37 °C for 30 min and terminated by adding of 200 µL of DNS reagent (1% 3,5-dinitrosalicylic acid and 12% sodium potassium tartrate in 0.4 M NaOH). The test tubes were then incubated in a boiling water bath for 5 min and cooled to room temperature. The absorbance was measured at 540 nm using a UV–Vis spectrophotometer. Acarbose was used as a positive control.

The results were expressed as percentage inhibition and calculated using the following formula:

inhibition (%) = [((Ac − Acb) − (As − Asb))/Ac − Acb] × 100

where Ac refers to the absorbance of control (enzyme and buffer); Acb refers to the absorbance of control blank (buffer without enzyme); As refers to the absorbance of sample (enzyme and inhibitor); and Asb is the absorbance of sample blank (inhibitor without enzyme). Also, the concentration of inhibitors required for inhibiting 50% of the α-amylase activity under the assay conditions was defined as the IC_50_ value.

#### 3.9.2. α-Glucosidase Inhibitory Assay

The α-glucosidase inhibitory activity of the extracts was measured following the method described by Kee et al. [55] with some modifications as reported by Asraoui et al. [56]. Briefly, samples were added to sodium phosphate buffer (pH = 6.7), mixed with 0.1 U/mL of α-glucosidase, and incubated at 37 °C for 10 min. One mM pNPG solution was then added to the mixture and incubated at 37 °C for 30 min. After this incubation step, 0.1 M of Na_2_CO_3_ was added and the absorbance was determined by spectrophotometer at 405 nm.

The α-glucosidase inhibitory activity was expressed as percentage inhibition, and the IC_50_ values were determined. Acarbose was used as a positive control.

### 3.10. In Vivo Antidiabituc Investigation

#### 3.10.1. Experimental Animals

Swiss Albino mice, male and female, aged 8 to 10 weeks and weighing between 23.5 and 28.5 g on average, were utilized in this investigation. The mice were housed in standard laboratory conditions, maintaining a constant room temperature of 20 ± 2 °C with a 12 h light–dark cycle. They were provided ad libitum access to standard rodent food and tap water prior to their involvement in the study [57].

#### 3.10.2. Diabetes Induction and Mice Grouping 

Mice were intraperitoneally injected with alloxan monohydrate at a dose of 180 mg/kg body weight, solubilized in cold PBS (0.1 M; pH 5), after 18 h of fasting with free access to water. After 4 days, a measurement of the blood glycemia of each mouse was performed. All mice with a blood glycemia level above 200 mg/dL were considered diabetic and designated for the study [58].

The mice were divided into five groups (Grp), each consisting of 6 mice; Grp 1 (normal): normal mice treated with distilled water. Grp 2 (diabetic-NT): diabetic mice treated with distilled water. Grp 3 (SE1-treated mice): diabetic mice treated with sorghum ecotype 1 seed at 125 mg/kg and Grp 4 (SE1-treated mice): diabetic mice treated with sorghum ecotype 1 seed at 250 mg/kg. Group 5 (glibenclamide-treated mice): diabetic mice treated with glibenclamide at 10 mg/kg. All mice were treated by oral gavage once a day for four weeks.

#### 3.10.3. Body Weight Assessment and Fasting Blood Glucose Level Measurement

Body Weight Assessment: Mice’s body weights were recorded at regular 7-day intervals throughout the experimental period using a digital weighing scale calibrated to the nearest gram. Measurements were taken in the morning before feeding to minimize variations due to food intake.

Fasting Blood Glucose Level Measurement: Blood glucose levels were measured after 12 h of fasting on days 0 to 21 of the experimental period. Briefly, mice were restrained gently, and a small incision was made at the tip of the tail. Blood samples were collected using a sterile lancet, and glucose levels were determined immediately using a glucometer (Accu-Chek, Roche Diagnostics, Mannheim, Germany) following the manufacturer’s instructions. Measurements were taken in triplicate, and the mean value was recorded for each animal at each time point [59].

#### 3.10.4. Collection of Blood Samples and Organs

Upon completion of the study, mice were anesthetized using ether and then euthanized to facilitate blood collection via cardiac puncture. Blood samples were subsequently transferred to tubes devoid of anticoagulant and allowed to stand at room temperature for 30 min. Following this, the tubes underwent centrifugation at 3000× *g* for 15 min at 4 °C to separate the supernatant, which was carefully preserved for serum parameter analysis [60]. Additionally, the liver and kidney were procured from each mouse, and the corresponding tissues were used for further biochemical assays.

#### 3.10.5. H_2_O_2_ and Malondialdehyde (MDA) Content Determination

H_2_O_2_ and malondialdehyde (MDA) contents were estimated according to Bouchmaa et al. [60,61].

#### 3.10.6. Antioxidant Enzyme Activity

Superoxide dismutase (SOD), catalase (CAT), glutathione peroxidase (GPx), and glutathione reductase (GR) activities were conducted according to our previous work [62].

### 3.11. Protein Content Assessment 

The amount of protein was measured using the Bradford method [63].

## 4. Conclusions

In the current study, a comprehensive analysis uncovering the intricate phenolic compound profile of sorghum seeds, unveiling a plethora of bioactive constituents with profound health implications, was reported. Procyanidin B-1 emerged as a standout contributor among phenolic compounds, renowned for its robust antioxidant prowess. 

The work delved into the ecotype-specific variations, shedding light on the distribution patterns of phenolic compounds across different sorghum ecotypes, highlighting the need for further investigations to unravel the underlying mechanisms driving these distinct profiles.

Moreover, sorghum’s therapeutic potential was assessed in diabetic mice demonstrating that a sorghum seed extract, viz., SE1 significantly reduces oxidative stress markers and enhances antioxidant enzyme activities in diabetic mice. These findings underscore the promising role of sorghum in managing diabetes-related complications and bolster its status as a promising candidate in food chemistry. Also, sorghum showed significant inhibitory activity against advanced glycation end products, particularly in the context of diabetes management.

Further research is warranted to fully elucidate the mechanisms underlying sorghum’s effects and optimize its therapeutic use in combating diabetes-related complications.

## Figures and Tables

**Table 1:** Characteristics of the compounds identified by HPLC-HRMS in the five ecotypes of sorghum seed ethanolic extract.

					Ecotypes				
Peak #	t_R_ (min)	[M + H]^+^	Name	Family	E1 (%)	E2 (%)	E3 (%)	E4 (%)	E5 (%)
1	0.7	171.0288	Gallic acid	I	0.5 ± 0.1 ^a^	0.5 ± 0.1 ^a^	0.4 ± 0.1 ^a^	-	-
2	1.1	169.0454	Vanillic acid	I	0.9 ± 0.2 ^a^	0.3 ± 0.1 ^b^	-	-	-
3	1.5	165.0546	p-coumaric acid	II	1.1 ± 0.2 ^a^	1.5 ± 0.2 ^a^	0.9 ± 0.1 a	1.0 ± 0.1 ^a^	-
4	3.8	155.03388	Protocatechic acid	I	1.7 ± 0.3 ^a^	2.9 ± 0.4 ^b^	1.0 ± 0.2 ^a^	1.0 ± 0.1 ^a^	0.8 ± 0.1 ^a^
5	5.1	579.1497	Procyanidin B-1	V	11.2 ± 1.1 ^a^	8.4 ± 0.9 ^b^	10.3 ± 1.0 ^a^	3.2 ± 0.3 ^c^	11.5 ± 1.1 ^a^
6	6.1	139.03897	Hydroxybenzoic acid	I	-	-	-	-	1.2 ± 0.2
7	6.1	291.0863	Catechin	V	53.8 ± 5.4 ^a^	40.8 ± 4.1 ^b^	54.6 ± 5.5 ^a^	19.6 ± 2.0 ^c^	68.6 ± 6.8 ^d^
8	7.0	181.0495	Caffeic acid	II	0.9 ± 0.1 ^a^	0.4 ± 0.1 ^b^	0.9 ± 0.1 ^a^	0.8 ± 0.1 ^a^	1.3 ± 0.1 ^a^
9	7.1	195.06518	Ferulic acid	II	1.3 ± 0.2 ^a^	1.3 ± 0.2 ^a^	0.6 ± 0.1 ^b^	0.5 ± 0.1 ^b^	0.7 ± 0.1 ^b^
10	8.2	289.07066	Eriodictyol	IV	5.6 ± 0.6 ^a^	6.7 ± 0.9 ^b^	2.4 ± 0.3 ^b^	1.0 ± 0.1 ^c^	-
11	9.1	305.06557	Taxifolin	V	6.8 ± 0.7 ^a^	9.7 ± 1.1 ^a^	7.9 ± 0.8 ^a^	38.4 ± 3.9 ^b^	3.3 ± 0.3 ^c^
12	9.4	273.07575	Naringenin	IV	1.9 ± 0.2 ^a^	2.4 ± 0.2 ^a^	0.8 ± 0.1 ^b^	0.5 ± 0.1 ^b^	-
13	9.5	271.0601	Apigenin	VI	2.3 ± 0.3 ^a^	4.3 ± 0.4 ^b^	4.5 ± 0.5 ^b^	5.7 ± 0.6 ^b^	1.9 ± 0.2 ^a^
14	10.6	303.04993	Quercetin	VI	0.2 ± 0.1 ^a^	2.3 ± 0.3 ^a^	1.9 ± 0.2 ^a^	9.5 ± 1.0 ^b^	0.7 ± 0.1 ^b^
15	10.6	611.16066	Rutin	IV	1.6 ± 0.2 ^a^	2.3 ± 0.3 ^b^	1.1 ± 0.1 ^a^	-	1.7 ± 0.2 ^ab^

Letters after the values indicate significantly different groups according to the statistical analysis ANOVA followed by Tukey HSD post hoc test. Values are expressed as mean ± SD (standard deviation); I: Hydroxybenzoic acids; II: Hydroxycinnamic acids; IV: Flavanones and Flavanols; V: Dihydroflavonols; VI: Flavones and Flavan-4-ol.

**Table 2:** Major and trace elements determined by inductively coupled plasma coupled with optical emission spectroscopy (ICP-OES).

Ecotype	Macroelements (mg/kg)					Microelements (mg/kg)			
	Ca	K	Mg	P	S	Fe	Mn	Si	Zn
E1	199 ± 0.5	510 ± 2.0	260 ± 3.0	530 ± 2.5	100 ± 1.6	57 ± 0.3	28 ± 0.2	396 ± 5.9	33 ± 0.6
E2	78 ± 0.5	300 ± 6.0	100 ± 0.9	220 ± 0.9	100 ± 1.2	30 ± 0.4	13 ± 0.1	408 ± 1.7	14 ± 0.1
E3	64 ± 0.8	240 ± 4.0	80 ± 0.7	180 ± 1.4	100 ± 0.9	18 ± 0.2	8.7 ± 0.02	375 ± 5.2	12 ± 0.3
E4	104 ± 0.4	250 ± 3.0	120 ± 0.9	250 ± 2.3	120 ± 1.3	33 ± 0.2	16 ± 0.1	472 ± 12.1	20 ± 0.3
E5	125 ± 0.8	530 ± 4.2	170 ± 5.0	420 ± 1.4	100 ± 0.5	195 ± 2.2	22 ± 0.1	361 ± 5.7	23 ± 0.9

**Table 3:** Ultra-trace elements determined by inductively coupled plasma coupled with mass spectrometry (ICP-MS).

Ecotype	^11^B (mg/Kg)	^111^Cd (µg/kg)	^59^Co (µg/kg)	^52^Cr (µg/kg)	^63^Cu (mg/Kg)	^95^Mo (mg/Kg)	^23^Na (mg/Kg)	^60^Ni (mg/Kg)	^208^Pb (µg/kg)	^78^Se (µg/kg)	^51^V (µg/kg)
E1	1.62 ± 0.03	<2	33 ± 1.38	204 ± 3.87	4.41 ± 0.04	0.52 ± 0.02	9.80 ± 0.22	0.45 ± 0.01	121 ± 1.69	12 ± 1.44	70 ± 1.58
E2	1.09 ± 0.04	<2	16 ± 1.04	192 ± 3.07	1.91 ± 0.04	0.380 ± 0.003	3.28 ± 0.22	0.340 ± 0.005	30 ± 0.72	15 ± 1.44	41 ± 2.04
E3	1.58 ± 0.05	<2	12 ± 0.84	68 ± 1.97	2.22 ± 0.02	0.70 ± 0.01	4.83 ± 0.60	0.156 ± 0.002	29 ± 0.66	16 ± 1.71	16 ± 1.38
E4	2.15 ± 0.03	<2	24 ± 0.98	249 ± 2.49	2.65 ± 0.05	0.96 ± 0.03	2.98 ± 0.10	0.56 ± 0.02	30 ± 0.63	23 ± 2.71	43 ± 2.92
E5	1.48 ± 0.04	<2	98 ± 2.54	688 ± 10.32	1.36 ± 0.01	0.61 ± 0.01	2.12 ± 0.02	0.67 ± 0.01	207 ± 3.72	17 ± 1.34	397 ± 10.95

**Table 4:** Polyphenol, flavonoid, and tannin content of sorghum ecotype extracts.

Ecotypes	Extract Yield (%)	Polyphenols (TPC) (mg GAE/g dE)	Flavonoids (TFC) (mg QE/g dE)	Tannins EAT (mg QE/mg dE)
E1	26.73 ^b^	297 ± 0.008 ^b^	72 ± 0.002 ^a^	0.253 ± 0.002 ^a^
E2	17.05 ^a^	188 ± 0.006 ^a^	66 ± 0.005 ^a^	0.210 ± 0.009 ^b^
E3	22.49 ^a^	229 ± 0.006 ^a^	78 ± 0.002 ^a^	0.203 ± 0.002 ^c^
E4	25.23 ^a^	262 ± 0.005 ^a^	73 ± 0.001 ^a^	0.222 ± 0.003 ^d^
E5	19.12 ^a^	192 ± 0.005 ^a^	70 ± 0.001 ^a^	0.195 ± 0.001 ^e^

Values are mean ± standard deviation. Statistical groupings are based on Tukey’s HSD test. Values followed by the same letter within a column are not significantly different (*p* > 0.05). GAE: Gallic Acid Equivalent. QE: Quercetin Equivalent. dE: dry extract.

**Table 5:** Antioxidant activities of sorghum ecotype ethanolic extracts.

Ecotypes	Antioxidant Properties (IC_50_ Values; mg/mL)			
	DPPH Scavenging Activity	ABTS	Metal Chelating Activity	Reducing Power (mg AAE/g dE)
E1	0.059 ± 0.002 ^a^	0.090 ± 0.010 ^a^	2.57 ± 0.10 ^a^	111 ± 8 ^a^
E2	0.060 ± 0.001 ^a^	0.131 ± 0.003 ^b^	1.816 ± 0.07 ^b^	121 ± 8 ^b^
E3	0.059 ± 0.002 ^a^	0.118 ± 0.002 ^b^	1.3 ± 0.1 ^b^	119 ± 3 ^b^
E4	0.043 ± 0.004 ^b^	0.118 ± 0.020 ^b^	2.110 ± 0.007 ^a^	101 ± 7 ^c^
E5	0.048 ± 0.001 ^b^	0.140 ± 0.010 ^b^	1.297 ± 0.020 ^b^	90 ± 3 ^c^
Trolox	0.203 ± 0.020 ^c^	0.086 ± 0.001 ^c^	-	-
EDTA	-	-	0.189 ± 0.030 ^c^	-

All values are mean ± standard deviation. Different letters indicate significant differences (ANOVA, Tukey’s test, *p* < 0.05) between conditions in the same test. IC_50_: the extract concentration providing 50% inhibition; DPPH: 2.2-diphenyl-1-picrylhydrazyl; ABTS: 2.2′-azino-bis (3-ethylbenzothiazoline-6-sulphonic acid); AAE: Ascorbic acid Equivalent; dE: dry extract.

**Table 6:** Inhibition results of sorghum-E extract on both α-amylase and α-glucosidase enzymes.

Ecotypes	IC50 (mg/mL)	
	Alpha-Amylase	Alpha-Glucosidase
Sorghum-E1	1.02 ± 0.20 ^a^	2.013 ± 0.008 ^a^
Sorghum-E2	1.54 ± 0.03 ^a^	1.07 ± 0.06 ^a^
Sorghum-E3	2.87 ± 0.01 ^a^	1.038 ± 0.040 ^a^
Sorghum-E4	3.4 ± 0.1 ^a^	1.889 ± 0.020 ^a^
Sorghum-E5	2.92 ± 0.06 ^a^	2.009 ± 0.020 ^a^
Acarbose	0.05 ± 0.02 ^b^	0.399 ± 0.003 ^b^

Values are means ± standard deviation. Different letters in the same column indicate significant differences (*p* < 0.05) according to Tukey’s multiple range test. For example, values followed by the same letter are not significantly different, while those followed by different letters are significantly different. Comparing the IC^50^ values of sorghum-E extracts to acarbose, a known α-glucosidase inhibitor used as a positive control, revealed that sorghum-E extracts from all ecotypes exhibited inhibitory activity against both enzymes, albeit with varying potency. For instance, sorghum-E1 and sorghum-E2 extracts displayed IC_50_ values of 1.021 mg/mL and 1.536 mg/mL, respectively, against α-amylase, indicating potent inhibition. Similarly, sorghum-E2, sorghum-E3, and sorghum-E5 extracts exhibited IC_50_ values ranging from 1.038 mg/mL to 1.889 mg/mL against α-glucosidase, demonstrating significant inhibitory activity.

**Table 7:** Effect of sorghum seed extract ecotype 1 on blood glucose level and body weight of alloxan-induced diabetic mice.

Treatment	Blood Glucose Level (mg/dL)				Body Weight (g)			
	Time (By Weeks)				Time (By Weeks)			
	Week 0	Week 1	Week 2	Week 3	Week 0	Week 1	Week 2	Week 3
Normal	78.01 ± 3.2 ^c^	70.00 ± 3.03 ^e^	77.65 ± 1.2 ^c^	73.43 ± 5 ^d^	21.12 ± 1.87 ^b^	25.02 ± 4.02 ^b^	22.91 ± 4.3 ^b^	23.12 ± 0.23 ^b^
Diabetic.NT	501 ± 9.00 ^a^	508.02 ± 32.0 ^a^	467.71 ± 43.9 ^a^	421.76 ± 26.7 ^a^	19.23 ± 3.01 ^a^	17.32 ± 2.3 ^a^	18.12 ± 3.05 ^a^	16.88 ± 3.93 ^a^
+125 mg/Kg	532 ± 54 ^a^	433 ± 12 ^b^	389.02 ± 65.0 ^b^	287.89 ± 42 ^b^	18.87 ± 3.87 ^a^	18.23 ± 4.02 ^a^	17.91 ± 3.4 ^a^	19.54 ± 1.05 ^a^
+250 mg/Kg	356.9 ± 28 ^b^	350.7 ± 65.1 ^c^	277.7 ± 23.7 ^b^	165.54 ± 9.02 ^c^	17.01 ± 3.65 ^a^	17.65 ± 3.22 ^a^	17.13 ± 4.06 ^a^	19.66 ± 1.24 ^a^
Glibenclamide	444.83 ± 43.02 ^a^	384.30 ± 54.3 ^d^	282.02 ± 17.8 ^b^	199.76 ± 23.1 ^c^	21.76 ± 4.3 ^a^	20.22 ± 2.43 ^a^	19.83 ± 3.90 ^a^	20.94 ± 3.46 ^a^

The data are presented as mean ± SEM. Statistical analysis was performed using one-way ANOVA followed by Tukey’s post hoc test for multiple comparisons. Different letters indicate statistically significant differences among the treatments (*p* < 0.05).

**Table 8:** Description of the 5 studied sorghum seed ecotypes.

Abbreviations of the Studied Ecotypes	Extraction Solvent	Designation of Sorghum Ecotype	Groups	Number of Ecotypes Per Group
E1	Ethanol	1P12	Gr4	5
E2	Ethanol	5P3	Gr1	2
E3	Ethanol	3P4	Gr4	5
E4	Ethanol	3P9	Gr3	2
E5	Ethanol	4P1	Gr2	1

**Table 9:** ICP-MS Agilent 7800 operating conditions.

ICP-MS Agilent 7800 Operating Conditions			
Collision/reaction cell gas	-	He	H_2_
Collision/reaction cell gas flow rate mL.min^−1^	-	4.3	4.2
Energy discrimination (V)	5	3	5
Nebulizer gas flow rate L.min^−1^		0.99	
Auxiliary gas flow rate L.min^−1^		0.9	
Plasma gas flow rate L.min^−1^		15	
Nebulizer type		Concentric Micromist	
Spray chamber		Quartz Scott double pass	
Injector		Quartz 2.5 mm	
RF Power (W)		1550	
Wash (s)		120	
Integration time (s)		1	
Replicates		4	
Sweeps		100	

## Data Availability

Data will be available upon reasonable request.

## Appendix A

**Table A1:** Morpho-agronomic traits evaluated for 10 Moroccan sorghum ecotypes.

Traits (Units)	Abbreviation	Method
Plant height (cm)	PH	Length in cm from base of the stalk to the tip of the panicle at maturity
Width of third leaf	WTF	Width of third leaf from top
Length of third leaf	LTF	Length of third leaf from top
Days to flag leaf appearance	DFL	Number of days from sowing to flag leaf appearance
Width of flag leaf	WFL	Width in cm at widest part of flag leaf
Length of flag leaf	LFL	Length in cm from base of flag leaf to leaf tip
Number of internodes	(NI)	Number of internodes from the base of the plant to the base of panicle
Diameter of third internode (cm)	DTI	Diameter of third internode from top was measured with calipers
Length of third internode (cm)	(LTI)	Length in cm of the third internode from top
Peduncle length (cm)	PL	Length in cm from ligule of flag leaf to base of panicle
Peduncle exertion (cm)	EX	Length of peduncle
Panicle length (cm)	PANL	Length in cm from the first whorl of branches to the tip of the rachis (maturity)
Panicle width (cm)	PW	Diameter at broadest part of panicle in cm
Number of primary branches per panicle	NPBP	The number of ramifications produced from the central axes of panicle
Peduncle shape	PS	These traits were observed according to Harlan and de Wet [64]. Briefly, the peduncle shape varies from erect to bent. Panicle compactness varies from loose to compact. Grain covering is the percentage of grain covered by glume. Grain shape can be rounded oval, or elliptic. Grain size is length of the longest axis of seed. Grain and glume color vary from white to brown.
Panicle compactness	PC	
Grain covering	GCOV	
Grain shape	GSH	
Grain size	GSI	
Grain color	GCOL	
Glume colour	GLC	
Days to flowering (days)	NDF	Number of days from planting to flower emergence
Weight of 100 grains (g)	HGW	Weight of 100 grains with grain humidity less than or equal to 12%; Based on random sample of 100-seeds taken four times from the bulked seeds of each experimental unit
Seedling vigor %	GV	Visual score of seedling growth at 20 days after sowing on a scale of 1 to 5, 1 being low vigor and 5 representing high vigor
Germination percentage %	GP	A seed is considered germinated when radical emerged by about 2 mm in length [65]
